# The cost of communication in the brain

**DOI:** 10.7554/eLife.27894

**Published:** 2017-05-23

**Authors:** Brian A MacVicar, Leigh Wicki-Stordeur, Louis-Philippe Bernier

**Affiliations:** Djavad Mowafaghian Centre for Brain Health, University of British Columbia, Vancouver, Canadabmacvicar@brain.ubc.ca; Djavad Mowafaghian Centre for Brain Health, University of British Columbia, Vancouver, Canada; Djavad Mowafaghian Centre for Brain Health, University of British Columbia, Vancouver, Canada

**Keywords:** ATP, axon, myelin, imaging, metabolism, brain, Mouse

## Abstract

Imaging ATP in axons reveals that they rely on glucose from the blood and lactate produced by glial cells as sources of energy.

**Related research article** Trevisiol A, Saab AS, Winkler U, Marx G, Imamura H, Möbius W, Kusch K, Nave K-A, Hirrlinger J. 2017. Monitoring ATP dynamics in electrically active white matter tracts. *eLife* **6**:e24241. doi: 10.7554/eLife.24241

Although metabolism is considered to be a dull subject by some, it is vital to life. The metabolic processes that convert food into energy are particularly important for the brain: although it accounts for just 2% of total body weight, the brain is responsible for 20% of the body’s total energy expenditure. Most of this energy comes from adenosine triphosphate (or ATP for short), which the body produces by metabolizing glucose and oxygen.

The neurons in the brain are made up of four distinct parts: the dendrites, which receive information from other neurons at special structures called synapses; the cell body, where the nucleus and genetic material are located; the axon, which carries information away from the cell; and the synapses at the end of the axon, where information is passed on to the dendrites of other neurons. The cell bodies, dendrites and synapses are located in the gray matter of the brain, while axons make up the white matter.

To use an analogy that may soon be redundant in the era of cell phones, axons are to neurons what telephone cables are to land-line phones. Like telephone cables, axons transmit information over long distances as electrical signals called action potentials (which are based on differences in the concentrations of certain ions inside and outside the neuron). There is usually a small voltage across the membrane of a neuron called a resting membrane potential. However, when a neuron is stimulated, various ions suddenly travel into or out of the neuron, changing this voltage and creating an action potential that travels along the axon. The efficiency with which information is communicated over long distances by complex networks is an impressive feature of our brains.

In humans, the white matter accounts for 50% of the brain volume (Laughlin and Sejnowski, 2003). The first signs of neurodegenerative diseases in the brain can often be seen in axons; a specific loss of axons in the white matter affects brain functions such as memory or vision, even though the cell bodies of neurons may still be intact. To better understand neurological diseases associated with degenerating axons and white matter lesions (Iadecola, 2013; Hirrlinger and Nave, 2014), it is important to quantify the amount of energy needed to fuel action potentials and axonal activity. Previous estimates based on glucose uptake measurements indicate that white matter consumes only one third of what gray matter requires.

Mathematical models suggest that the gray matter requires a lot of energy for synaptic transmission, which involves molecules called neurotransmitters traveling from a pre-synaptic neuron to a post-synaptic neuron (Attwell and Laughlin, 2001). Most of the energy in the white matter, however, is used for generating an action potential and subsequently reestablishing the resting membrane potential (Harris and Attwell, 2012). Yet, the energy use of the white matter is still poorly understood, as it has been difficult to measure the exact amount of ATP consumption during the generation of action potentials in the axons.

Now, in eLife, Johannes Hirrlinger, Klaus-Armin Nave and colleagues – including Andrea Trevisiol of the Max Planck Institute for Experimental Medicine as first author – report that they have used optical sensors to directly measure the ATP consumption required to power action potentials (Trevisiol et al., 2017) This new approach allowed them to do two things for the first time: to visualize the energy use of action potentials in real time, and to determine the metabolic source of the ATP. The experiments were performed on an adapted version of the mouse optic nerve, a well-established nerve model for white matter electrophysiology (Stys et al., 1991; Brown et al., 2003).

Trevisiol et al. first uncovered a remarkable correlation between ATP levels and the generation of action potentials. When action potentials were evoked more frequently, the ATP levels decreased, indicating that action potentials rapidly consume energy. On the other hand, when the ATP production was interrupted, action potentials in the axons declined and progressively failed, because they did not spread as effectively. However, the technique cannot determine the exact levels of energy consumption because their optical sensor cannot measure the absolute concentration of ATP.

Trevisiol et al. subsequently showed that axons rely on several sources of energy for ATP production. Although glucose from the blood is the principal source, the researchers were able to show that the axons also use lactate as an energy source. It is thought that glial cells called astrocytes, which are found in both white and gray matter, metabolize a form of glucose called glycogen to produce lactate (Brown et al., 2003; Suzuki et al., 2011). Other glial cells called oligodendrocytes can also supply lactate (which they produce by metabolizing glucose) (Fünfschilling et al., 2012; Lee et al., 2012). This suggests that a complex energy supply network in which multiple cell types and metabolic energy sources are used to maintain the ATP levels is crucial for axons to work properly.

This development of an imaging approach that can monitor changes in ATP levels is an important step in quantifying the metabolic costs of communication via white matter axons. It also gives us a clever insight into defining the important supporting roles of glia cells in maintaining the health of the white matter itself via the production of lactate.
